# The effect of a universal mobile application on adolescents' mental health and well-being

**DOI:** 10.1016/j.invent.2025.100814

**Published:** 2025-02-18

**Authors:** Helene Høgsdal, Sabine Kaiser, Geraldine Mabille, Monica Martinussen, Reidar Jakobsen, Henriette Kyrrestad

**Affiliations:** a Regional Centre for Child and Youth Mental Health and Child Welfare - North, Faculty of Health Sciences, UiT The Arctic University of Norway, Tromsø, Norway; b Department of Clinical Psychology, University of Bergen, Bergen, Norway

**Keywords:** Adolescents, Mental health, Mental health app, Mental health promotion, Opp, Well-being

## Abstract

Opp is a universal mental health-promoting mobile application (app) developed for adolescents, with the aim of promoting mental health and well-being and preventing mental health problems. In this randomized controlled trial, the effectiveness of Opp was tested among Norwegian adolescents aged 13 to 25 years. Mental health, well-being, self-efficacy, self-esteem, help-seeking behavior, and sleep quality were assessed at two measurement points (T1 and T2), that were approximately 11 weeks apart. Only the participants that answered at both measurement points were included in the main analyses (*N* = 399; 75 % girls; *Mage* = 16.90 years, *SD* = 1.40). The results demonstrated a statistically significant effect of the app on mental health, as measured by the SDQ Total Difficulties scale (*F*(1,790) = 4.35, *p* = .037), while no statistically significant effects were observed on the other outcomes. These results provide important insights, and a broader understanding of how mental health apps can influence the mental health and well-being of a general sample of adolescents.

## Highlights

• Opp is a mobile app that aims to increase mental health and well-being among adolescents.
• The effectiveness of the app was evaluated in a randomized controlled trial with two measurement points.
• Results showed an increase in mental health problems in the control group, but not in the intervention group.
• Although the findings are promising, more research is needed to understand the potential of universal mental health apps for adolescents.

## 1 Introduction

Adolescence is a unique time, stretching from childhood and into adulthood (Sawyer et al., 2018). Rapid and complex changes occur during this time period, and the risk of developing mental health problems increases (Barican et al., 2022; Blakemore, 2019; Yoon et al., 2023). In Norway, self-reported mental health problems among adolescents have increased during the last decade, especially among girls (Bakken, 2024; Knapstad et al., 2021). This indicates that many adolescents are in need of support and mental health care. At the same time, evidence suggests that many young people do not receive help due to a lack of resources within healthcare systems (Canady, 2023; Mojtabai et al., 2016; Rocha et al., 2015). Thus, developing mental health-promoting interventions that support adolescents and strengthen their competence, resilience, and empowerment should be a priority (Barry et al., 2019; Blakemore, 2019; Bor et al., 2014).

The World Health Organization (WHO, 2020) states that universal mental health interventions should include strategies for emotion regulation, mindfulness, and stress management. Furthermore, these interventions should be implemented in arenas where they can impact adolescents' lives (e.g., in schools; Jané-Llopis and Barry, 2005; O'Reilly et al., 2018). Adolescents' frequent interaction with technology presents opportunities to offer interventions tailored to their needs on digital platforms. Specifically, mobile applications (apps) have been highlighted as a promising platform to deliver cost-effective and easily accessible mental health interventions (Donker et al., 2013; Grist et al., 2017; Olff, 2015).

### 1.1 Mental health promoting mobile applications

Mental health apps can be designed to target specific mental disorders, such as anxiety (Sucala et al., 2017). Alternatively, mental health apps can provide psychoeducation and other resources such as emotion regulation strategies (Kaiser et al., 2022; Olff, 2015), with the goal of serving as universal tools for promoting mental health among their users. While there is some evidence suggesting that mental health apps can be promising tools for improving outcomes such as anxiety and depression (Lehtimaki et al., 2021), there are still uncertainties regarding their impact on broader outcomes such as well-being and general mental health (Lehtimaki et al., 2021; Litke et al., 2023). Adolescents are often satisfied with their experience using mental health apps, and often report positive feedback on apps' usability and acceptability (Bear et al., 2024; Grist et al., 2017). At the same time, it has been revealed that engaging adolescents with mental health apps is challenging in terms of both initial engagement and ongoing use (Bear et al., 2024; Leech et al., 2021). This illustrates the importance of involving adolescents in the development process of mental health apps intended for their use, to ensure that the apps are developed according to their specific needs and preferences (Bear et al., 2024). Additionally, there is a need for more research on the mental health apps that are developed in order to establish their actual efficacy and validate their use as reliable tools for mental health support among adolescents (Bear et al., 2024; Grist et al., 2017; Leech et al., 2021).

### 1.2 Opp – A universal mental health promoting mobile app

Opp is a mobile app developed with the goal of improving mental health and preventing the development of mental health problems among adolescents. In addition, the app also aims to increase self-efficacy, self-esteem, sleep and help-seeking behavior (Kaiser et al., 2022). Opp was developed together with Norwegian adolescents and a reference group consisting of researchers, social workers, health nurses and psychologists working with youth and mental health. In workshops that were conducted prior to the development of the app, both the adolescents and members of the reference group contributed with ideas on what the app should contain and how it should appear in terms of concept, design and terminology. Furthermore, two adolescents were involved while the app was being developed, providing feedback on whether the information presented in the app was appropriate for the target group (see Kaiser et al., 2022 for more details on how the app was developed).

**Fig. 1:**
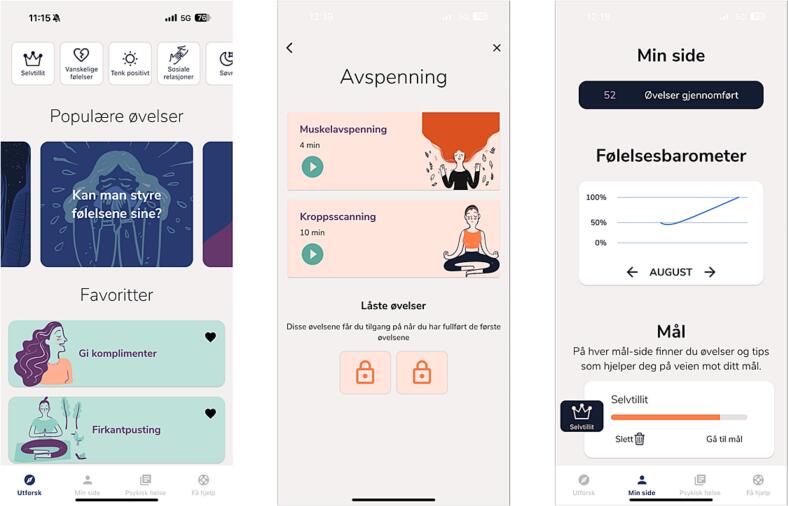
Screenshots of Opp.

The app consists of a psychoeducation module and a resource module (See Fig. 1 for three screenshots of Opp). The psychoeducation module includes information about mental health (e.g., what is mental health, why is mental health important and how to obtain and maintain good mental health). This module also includes information about a range of emotions adolescents often experience (e.g., feeling in love, happiness, loneliness, sadness) and information about where and how to seek mental health support. The resource module consists of exercises and techniques on how to cope with challenging thoughts, feelings and stress (e.g., breathing techniques, sleep advice and relaxation exercises). The resource module also includes an exercise, based on cognitive behavioral therapy, called “thought clearing”, which aims for the user to recognize and challenge negative thoughts. The app also contains a separate “My page”, where app users can set their own goals (e.g., improve self-esteem), track how many exercises they have completed, and see how their mood has developed over the past weeks in a graph. A user survey of Opp indicated that young people in the target group assessed the app to be of good quality, and to have acceptable usability (Høgsdal et al., 2023).

### 1.3 The present study

The purpose of this study is to evaluate the effectiveness of Opp on its primary (mental health and well-being) and secondary (i.e., self-efficacy, help-seeking behavior, self-esteem measured by the two dimensions self-liking and self-competence, and sleep) outcomes among a sample of adolescents, using a randomized controlled trial design. We hypothesized that access to Opp would lead to significant changes in mental health, well-being, self-efficacy, self-esteem, sleep, and help-seeking behavior among the participants.

## 2 Methods

### 2.1 Study design

The study has a between-subjects experimental design (intervention condition vs. waiting-list control condition; ClinicalTrials.gov NCT05211713). Initially, the study design was intended to follow a cluster randomized controlled trial with three measurement points (pre-test, post-test, and follow-up), where the individuals would be randomized at the school level (Kaiser et al., 2022). However, due to the low number of participants who signed up for the study, the design was changed to only include two measurements points (pre-test [T1] and post-test [T2]), and participants were randomized at the individual level.

### 2.2 Recruitment and procedure

Participants were recruited during 2022 and 2023. Principals, school health nurses, and other school employees in secondary schools, high schools, folk high schools, and universities in Norway were informed about the study and asked to share the information and a digital consent form with their students (Nettskjema, 2024). In line with requirements from the Regional Committees for Medical and Health Research Ethics (REK North; ref. 279207), participants older than 16 years could consent by themselves, while active parental consent from all guardians was required for the participants younger than 16 years.

Participants received a SMS or an email with a link to the first questionnaire (T1). After answering T1, a statistician randomized participants into the intervention or waiting-list control group. Participants in the intervention group received an SMS, with a code to download and open the app in addition to a reminder to use and to engage with the app after one week. After six weeks, all participants received a link to the post-test questionnaire (T2). However, due to a long response time from the adolescents, the response time between T1 and T2 was approximately 11 weeks. Participants who answered both questionnaires were entered into a random drawing for 100 gift cards of 300 NOK each (approximately 25 USD).

### 2.3 Measures

#### 2.3.1 Demographic characteristics

Demographic information included age (13, 14, 15, etc. to 20 years or older) and sex (male, female).

#### 2.3.2 Mental health and well-being

Participants under the age of 18 answered the Strength and Difficulties Questionnaire self-report (SDQ—S; Goodman and Goodman, 2009) while participants older than 18 years answered the self-report questionnaire adapted to those aged 18 and over (SDQ-S18+; www.sdqinfo.org). The two versions of the questionnaires are similar but have slight differences in the wording of some questions (e.g., “I usually share with others, for example CD's, games, food” [SDQ—S] versus “I usually share with others, for example food or drink” [SDQ—S18+]; Brann et al., 2018). Both versions consist of 25 items, constituting four problem-subscales and one subscale measuring prosocial behavior (e.g., “I try to be nice to other people. I care about their feelings”). The four problem-subscales measure 1) Emotional problems (e.g., “I worry a lot”); 2) Conduct problems (e.g., “I get very angry and often lose my temper”); 3) Hyperactivity/inattention problems (e.g., “I am easily distracted, I find it difficult to concentrate”); and 4) Peer problems (e.g., “I get along better with adults than with people my own age”). Combined, the four problem subscales also contribute to the Total Difficulties Scale. Responses are rated on a 3-point Likert scale from 0 to 2 (0 = Not true, 1 = Somewhat true, 2 = Certainly true). The SDQ has been translated and used in Norwegian studies and has shown varying degrees of internal consistency (Kornør and Heyerdahl, 2013). Cronbach's alphas based on the current sample were as follows: the Total Difficulties Scale T1 α = 0.80; T2 α = 0.83; Emotional Problems T1 α = 0.76; T2 α = 0.79, Conduct Problems T1 α = 0.51; T2 α = 0.53, Hyperactivity/inattention T1 α = 0.75; T2 α = 0.78, Peer Relationship Problems T1 α = 0.63; T2 α = 0.62 and Prosocial Behavior: T1 α = 0.65; T2 α = 0.66.

The World Health Organization Well-Being Index (WHO-5) was used to measure psychological well-being. The WHO-5 consists of five positive phrased items (e.g., “Over the last two weeks I have felt calm and relaxed”). Responses are rated on a 6-point Likert scale from 0 to 5 (0 = At no time, 1 = Some of the time, 2 = Less than half of the time, 3 = More than half of the time, 4 = Most of the time, 5 = All of the time). In this study, all item scores were summed and converted to a score from 0 to 25, before being multiplied by 4 for the sum to range from 0 (absence of well-being) to 100 (maximal well-being; Topp et al., 2015). Cronbach's alphas were as follows: T1 α = 0.82; T2 α = 0.83.

#### 2.3.3 Self-efficacy

The General Self-Efficacy Scale (GSE; Schwarzer and Jerusalem, 1995) was used to measure general self-efficacy. The scale consists of ten items (e.g.,”I can always manage to solve difficult problems if I try hard enough”). Responses are rated on a 4-point scale (1 = Not at all true, 2 = Hardly true, 3 = Moderately true, 4 = Exactly true). The ten items were summed up to a total score ranging from 10 (low self-efficacy) to 40 (high self-efficacy). The scale has been translated and tested in Norwegian contexts and has been found to have satisfactory internal consistency and test-retest reliability (Leganger et al., 2000). Cronbach's alphas were as follows: T1 α = 0.89; T2 α = 0.90.

#### 2.3.4 Self–esteem

The Self–Liking and Competence Scale (Tafarodi and Swann Jr, 1995) was used to measure self-esteem among the participants. The questionnaire consists of 20 items, ten items measuring self-liking (e.g., “I feel comfortable with myself”) and ten items measuring self-competence (e.g., “Owning to my capabilities, I have much potential”). Both dimensions are considered to measure self-esteem, with self-liking revolving around a person's sense of social worth and self-competence revolving around a person's sense of personal efficacy (Tafarodi and Swann Jr, 1995). Responses are rated on a five-point scale, ranging from 1 (strongly disagree) to 5 (strongly agree). Both dimensions (i.e., self-liking and self- competence) are presented separately in this study. These scales have been validated in a Norwegian context and have demonstrated good internal reliability (Silvera et al., 2001). In the current study, Cronbach's alphas were as follows for self-liking: T1 α = 0.94; T2 α = 0.94, and for self-competence: T1 α = 0.93; T2 α = 0.93.

#### 2.3.5 Sleep

Sleep was measured with a question from the Bergen Child study (Hysing et al., 2018). The participants were asked to rate the following statement “I have trouble falling asleep and/or wake up frequently” on a 3-point scale (1 = True, 2 = Somewhat true, 3 = Not true).

#### 2.3.6 Help-seeking behavior

Help-seeking behavior was measured using the question “Have you ever had a hard time and told someone about what was bothering you, so that they could help you?”. Participants could respond “Yes” or “No”.

#### 2.3.7 Use of Opp

Participants in the intervention group were asked “How many times per week have you used the app?”. Responses were given on a scale ranging from 0 times, one time, two times, three to five times, six to nine times, ten to 13 times, and >13 times.

### 2.4 Analytic strategies

A power analysis conducted in PASS 16 estimated that 352 participants (176 in each group) were needed to detect an effect corresponding to a Cohen's *d* = 0.30 with a power = 0.80.

Descriptive statistics were calculated, including means, standard deviations and frequency distributions. Univariate general linear models were used to test for statistically significant differences between the groups at baseline. A logistic regression analysis was used to test for selective dropout among the participants. Participation at T2 was coded as a binary variable (1 = participation, 0 = dropout), and the baseline variables were included as predictors in the model.

Generalized linear mixed models with normal distributions were used to estimate the effect of Opp on the primary and secondary outcomes, except for help-seeking, which was analyzed using a binomial distribution. The analyses were conducted using an intention-to-treat approach (i.e., 2 conditions: intervention versus control group). However, a per-protocol approach was also conducted (i.e., 3 conditions: participants in the intervention group that had used the app versus participants in the intervention group that had not used the app versus participants in the control group; the results are presented in Table S1 and S2 in the supplementary materials). The models included the predictors age, sex, time (coded as a categorical variable: T1 and T2), condition, and an interaction term between condition and time. If there was no significant interaction between condition and time, we reran the models without the interaction to investigate condition and time as main effects. Computations of effect sizes (ES) were based on estimates obtained from the mixed models and were calculated as the differences in estimated marginal means between the intervention (*MT1i* – *MT2i*) and control (*MT1c-MT2c*) groups, divided by the pooled standard deviations (*SpT1*) for the total sample at T1 ($\frac{\mathrm{MT}1i-\mathrm{MT}2i-\mathrm{MT}1c-\mathrm{MT}2c}{\mathrm{SpT}1}$). SPSS version 29 was used for all analyses and a *p*-value <0.05 was considered statistically significant.

## 3 Results

### 3.1 Descriptives

The baseline sample consisted of 736 participants (72 % girls, *M*_*age*_ = 16.76 years, *SD* = 1.52). A total of 325 participants were allocated to the control group and 411 to the intervention group. A substantial proportion (46 %) of the baseline sample did not answer the questionnaire at T2. The attrition analysis revealed that older age, lower well-being, and higher help-seeking behavior increased the odds of participation at T2 (Table 1). Participants who did not respond at T2 were excluded from further analyses, resulting in a final sample of 399 participants (75 % girls; *M*_*age*_ = 16.90 years, *SD* = 1.40) of which 168 were in the control group, and 231 were in the intervention group. At baseline, the groups only differed in the WHO-5 scores; with the intervention group scoring higher on well-being compared to the control group (Table 2). In the intervention group, *n* = 59 (25.5 %) stated that they had used the app 0 times per week. The majority reported that they had used the app once (*n* = 111, 48.1 %) or twice (*n* = 37, 16.0 %) per week, while 20 participants (8.7 %) reported using the app 3 to 5 times per week, and 4 participants (1.7 %) reported using it more than five times per week.

**Table 1:** Attrition Analyses Predicting Participation at T2.

	*B*	SE	Wald	DF	*p*	EXP (*B*)
Age	0.11	0.05	4.19	1.00	**0.041**	1.11
Sex^a^	0.01	0.19	0.00	1.00	0.969	1.01
Condition^b^	0.15	0.16	0.95	1.00	0.329	1.17
Total Difficulties	−0.03	0.02	2.73	1.00	0.098	0.97
WHO-5	−0.02	0.01	9.70	1.00	**0.002**	0.98
Self-efficacy	0.02	0.02	1.13	1.00	0.288	1.02
Self-competence	−0.01	0.02	0.19	1.00	0.665	0.99
Self-liking	−0.01	0.01	0.19	1.00	0.661	0.99
Sleep	−0.12	0.11	1.28	1.00	0.258	0.88
Help-seeking^c^	0.53	0.17	9.73	1.00	**0.002**	1.69

a The reference category was female

b The reference category was control group

c The reference category was not sought help.

**Table 2:** Differences Between Groups at Baseline (*N* = 399).

	Control *n* = 168			Intervention *n* = 231			Total *N* = 399		
	*M*	*SD*	*%*	*M*	*SD*	*%*	*M*	*SD*	*%*
Age	17.01	1.42		16.83	1.38		16.90	1.40	
Gender: males			23.2			26.8			25.3
Total Difficulties	13.92	5.60		13.39	5.99		13.61	5.83	
**WHO-5**⁎	47.24	19.45		51.36	20.34		49.62	20.05	
Self-efficacy	18.72	5.78		19.19	4.94		18.99	5.30	
Self-competence	34.32	9.09		35.25	8.23		34.86	8.60	
Self-liking	26.40	9.67		26.68	9.21		26.56	9.40	
Sleep problems	1.96	0.85		1.81	0.82		1.87	0.84	
Help-seeking: sought help			63.9			60.6			62.0

Note.

⁎ *F*(1,398) = 4.14, *p* = .042.

### 3.2 Mental health and well-being

**Fig. 2:**
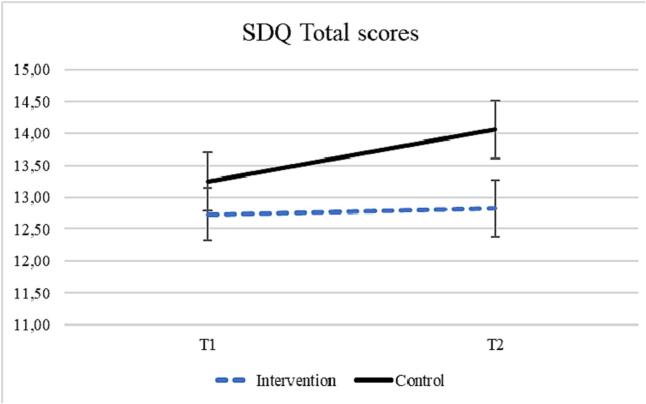
Note. The estimates are calculated for an average age of the sample (*M* = 16.90 years).

The interaction between time and condition was statistically significant in the model predicting the Total difficulties scale (*F*(1,790) = 4.35, *p* = .037, *ES* = 0.13). The Total difficulties scale increased over time among the participants in the control group, and not among participants in the intervention group (Fig. 2). There were no significant interaction effects in the models examining the different SDQ subscales (*p* > .079), or in the model predicting well-being (*p* = .729, See Table 3). There was a main effect of time for conduct problems- and peer problems scores at T2 (*F*(1,791) = 5.10, *p* = .024 and *F*(1,791) = 8.64, *p* = .003, respectively), indicating an increase in the scores at T2.

**Table 3:** Results from Mixed Effect Models Predicting Mental Health, Well-being Self-Efficacy, Self-Competence, Self-liking, Sleep Problems and Help-seeking Behavior.

	Total difficulties			Well-being			Self-efficacy			Self-competence			Self-liking			Sleep problems			Help-seeking behavior		
	*B*	SE	*p*	*B*	SE	*p*	*B*	SE	*p*	*B*	SE	*p*	*Β*	SE	*p*	*B*	SE	*p*	*B*	SE	*p*
Age	−0.39	0.21	0.064	0.49	0.66	0.457	0.20	0.18	0.259	0.36	0.28	0.209	0.12	0.32	0.696	−0.02	0.03	0.580	0.14	0.07	0.056
Sex^a^	−2.69	0.64	**<0.001**	12.57	1.99	**<0.001**	3.05	0.51	**<0.001**	3.56	0.89	**<0.001**	4.70	0.98	**<0.001**	−0.19	0.08	**0.022**	−1.32	0.24	**<0.001**
Time^b^	0.82	0.25	**0.001**	−0.22	1.23	0.861	−0.21	0.34	0.536	−0.85	0.42	**0.044**	−0.34	0.40	0.395	−0.04	0.05	0.409	−0.44	0.18	**0.017**
Condition^c^	−0.51	0.57	0.370	3.76	1.95	0.054	0.37	0.53	0.482	0.86	0.88	0.330	0.13	0.95	0.890	−0.15	0.08	0.077	−0.08	0.24	0.745
Time^b^ x Condition^c^	−0.73	0.35	**0.037**	−0.56	1.61	0.729	0.22	0.40	0.582	0.42	0.58	0.476	0.51	0.55	0.353	0.03	0.07	0.674	0.15	0.24	0.528

a female is the reference category.

b T1 is the reference category.

c control group is the reference category.

### 3.3 Self-efficacy, self-esteem, sleep and help-seeking behavior

There was no significant effect of the interaction terms between time and condition for self-efficacy, self-esteem (i.e., self-liking and self-competence), sleep or help-seeking behavior (all *p* > .353). There was a significant main effect of time on self-competence (*F*(1,789) = 4.37, *p* = .037), and help-seeking behavior (*F*(1,787) = 8.41, *p* = .004), indicating that self-competence and help-seeking behavior decreased during the study period (*B* = −0.61, *B* = −0.35, respectively).

## 4 Discussion

The aim of this study was to evaluate the effectiveness of Opp, a universal mental health promoting mobile application, on mental health and well-being outcomes in a general sample of young people aged 13–25 years.

Opp demonstrated a promising effect on mental health. The score on the Total difficulties scale increased (about one point) in the control group while no concurrent increase was observed in the intervention group. It is challenging to interpret this rapid increase of total difficulties in the control group during the study period. In Norway a score of up to 14 on the Total difficulties scale has been considered normal for adolescents (Kornør and Heyerdahl, 2013). While Goodman and Goodman (2009) argue that there is no specific score at which the likelihood of developing a mental disorder increases, they also report that each 1-point increase in the Total difficulties scale increases the odds of developing a mental disorder by 14 % to 28 %. Although it is well known that self-reported mental health problems increase throughout adolescence among Norwegian youth (Bakken, 2024), the overall increase in total difficulties in the control group was unexpected. One possible explanation is that many participants completed T2 during the winter months in Norway (median date: 4. December 2022), when seasonal factors such as reduced daylight can cause variations in mood (Friborg et al., 2012). Additionally, the median response time for T2 overlapped with the exam period in schools. Many Norwegian adolescents, especially girls, experience stress and pressure to do well in school, which can contribute to a deterioration in self-perceived mental health (Bakken, 2024; Eriksen, 2021). The participants who had access to Opp during the study period did not report an increase in mental health problems, suggesting that Opp may have a preventive effect on the development of such issues. This finding indicates that Opp could also be a valuable tool for professionals working with early prevention and universal mental health promotion, such as health care nurses.

The use of Opp did not influence the participants' well-being, self-efficacy, self-esteem and sleep. A possible explanation could be that the intervention is not effective on these outcomes, or that mental health apps in general do not have an effect on these outcomes when being tested in a general adolescent population. Regarding the well-being outcome, the CopeSmart app, which is similar to Opp, also showed no effect on well-being among a sample of adolescents (Kenny et al., 2020). On the other hand, the WeClick app had a short-term positive effect on young people's well-being (O'Dea et al., 2020). WeClick differs from Opp as it is an interactive story-telling app that targets relationship issues (e.g., family and peer conflicts; O'Dea et al., 2020). In the WeClick app, young people can choose a character and complete activities with the goal of learning to think more positively and solve problems by observing what happens in other people's relationships. In contrast, Opp is self-focused, meaning that its exercises are designed for adolescents to work on themselves, such as through breathing exercises. These results may suggest that incorporating other people's stories in apps could be a more effective way to enhance youth well-being. However, this assumption cannot be confirmed, as there may be other differences between the Opp and WeClick apps that could explain their varying effectiveness in enhancing well-being among adolescents. Additionally, there is limited evidence supporting the effectiveness of universal apps in promoting well-being among adolescents, indicating a need for further research (Leech et al., 2021). Comparing the findings on Opp's lack of effect on the participants' self-efficacy, self-esteem, and sleep was challenging, as research on the effectiveness of mental health apps for these outcomes is often conducted on adults or young people with mental health problems (Bruhns et al., 2021; Giraldo-O'Meara and Doron, 2021; Werner-Seidler et al., 2023).

Due to adolescents' negative beliefs towards mental health services (Aguirre-Velasco et al., 2020), mental health apps have been highlighted as a promising tool to increase help-seeking behavior because they offer anonymity and autonomy (Garrido et al., 2022; Høgsdal et al., 2024; Olff, 2015). Internet-based interventions have been found to be able to increase help-seeking behavior (Johnson et al., 2022). However, another study conducted by Wiljer et al. (2020) found that the mobile app Though Spot did not promote help-seeking behavior to a greater extent than other non-digital platforms. In Opp, the users are informed about *where* they can seek help, and *how* they can approach mental health services in Norway. Nonetheless, Opp did not contribute to increase help-seeking behavior among the participants, probably partly because providing information was not enough to encourage the young people to ask for help. Also, the mental health scores in the intervention group were stable during the study period, indicating that there might not have been a need to ask for help. Whether Opp can contribute to increase help-seeking behavior among young people if they experience increased mental health problems remains to be explored.

### 4.1 Strengths and limitations

A strength of the study is the use of a RCT design to investigate the effect of Opp. There is a need for studies evaluating mental health apps developed for adolescents, as knowledge about their effectiveness is limited (Litke et al., 2023). However, it is important to interpret the results from this study with caution. Recruiting participants to the study was challenging and nearly half of the participants that answered at baseline did not answer at post-test. Also, despite the assistance from school personnel in sharing enrollment information, few signed up to participate. It is possible that many adolescents were reluctant to participate because the information came from their school. Studies have shown that participants tend to be more motivated to engage in research involving mental health apps when the invitation comes directly from the researchers responsible for the study (Linardon and Fuller-Tyszkiewicz, 2020). Another factor that may explain why few participants signed up for the study is that we targeted a general population of adolescents who might not have perceived the value of testing a mental health promoting mobile app if they had no mental health difficulties. This assumption is supported by the attrition analyses, which indicate that, in addition to being female and of older age, having lower well-being, and higher help-seeking behavior predicted participation at T2. This suggests that testing a mental health-promoting app may be more valuable for those already experiencing lower well-being or greater difficulties. Additionally, individuals with a lower threshold for seeking help might also be more inclined to use such an app.

There is a lack of objective data on how much the participants used the app during the study period. The question “How often have you used the app each week” might have led to underreporting of app usage. The question was intended to assess the participants' actual app use, but it might have been too vague to accurately determine how much the app was used during the study period. That is, given the existing knowledge that adolescents often do not engage frequently with mental health apps (Bear et al., 2024; Leech et al., 2021), it is reasonable to assume that most of the participants did not use the app *each* week. For example, it is possible that participants that had used the app only a few times during the whole study period might have reported zero weekly usage, despite having engaged with the app at least occasionally. When analyzing the results from the per-protocol analyses, we found that the difference in the change in Total difficulties between the control and intervention groups, as identified in the intention-to-treat analyses, was mainly driven by a decrease in Total difficulties among the group that answered they had not been using the app weekly (see supplementary material). Those reporting that they had not been using the app each week were probably also those experiencing the least difficulties and experiencing less frequent need to use the app. This is also probably why they are those for which the difficulties score decreased the most. A more effective approach to measure actual app use could have been to ask participants whether they had used the app at all during the study period, with “yes” or “no” as response options. However, this method has also been found to complicate the accurate assessment of adolescents' actual app usage (Kenny et al., 2020). In general, a study by Boyle et al. (2022) indicated that adolescents' self-reported screen use is often inaccurate, underlining the need to use objective data when assessing adolescents' usage of mental health apps.

Another limitation is that single items were used to measure both sleep quality and help-seeking behavior. Thus, the reliability of these items is uncertain (Allen et al., 2022). Furthermore, two of the problem subscales in the SDQ (conduct and peer problems) had low internal consistency in this study. A psychometric review of the SDQ in a Scandinavian context revealed that these subscales often have an internal consistency similar to what we found in this study (Kornør and Heyerdahl, 2013). However, the SDQ is still recommended to be used when doing research on adolescents' mental health in Scandinavia (Kornør and Heyerdahl, 2013). Because all the other SDQ subscales, as well as the Total difficulties Scale, demonstrated acceptable internal consistency, all scales were included and presented in this study.

### 4.2 Future research

To better understand the preventive effects of universal mental health apps, the apps should be further tested on multiple samples in the general youth population. Furthermore, since app usage and app engagement are often low among adolescents (Bear et al., 2024; Leech et al., 2021), future research should incoporate appropriate methods to track their actual app use in a way that respects their user privacy. Additionally, investigating which modules of Opp were most frequently used would be beneficial. This information could be used to delete or improve the less used modules while the most used modules could be further developped to make the app even more attractive for adolescents.

## 5 Conclusion

This study evaluated the effectiveness of Opp – a universal mental health promoting mobile application. The results indicated a stagnation in the development of mental health problems among participants in the intervention group during the study period while those problems increased in the control group. This suggests that Opp may have a preventive effect against the development of mental health problems. Opp did not influence other mental health outcomes such as well-being, self-efficacy, self-esteem, sleep, or help-seeking behavior. Although Opp shows potential as a self-help tool for adolescents, further research should, to a greater extent, examine adolescents' actual usage of the app. By gaining a more thorough understanding of the adolescents' use of the app, one can also investigate whether higher use of the app can have an effect on the outcome measures that the app aims to improve among adolescents.

## Declaration of competing interest

The authors declare the following financial interests/personal relationships which may be considered as potential competing interests: Author HK reports that the development of Opp was funded by a grant from the Foundation Dam through the Norwegian Council of Mental Health (Ref. 2020HE2–328433). The authors SK, MM, RJ, and HK have contributed to the development of the mobile app. No financial interests are attached as the mobile app is available for free in Google Play and App Store in Scandinavia. If there are other authors, they declare that they have no known competing financial interests or personal relationships that could have appeared to influence the work reported in this paper.

## Appendix A Supplementary data

Supplementary material Image 1
