# Supplementary material for: The effect of a universal mobile application on adolescents' mental health and well-being
Source: Internet Interv. 2025 Feb 18;40:100814. doi: 10.1016/j.invent.2025.100814 (PMC11929045; doi:10.1016/j.invent.2025.100814)
Supplement: Supplementary file 1 — Supplementary material [file mmc1.docx]

**Supplementary material**

Table S1 – S2 contain the per-protocol (pp) analyses.

**S1 Table**

*Fixed effects for Mental health, Well-being, Self-efficacy, Self-competence, Self-liking, Sleep problems, Help-seeking Behavior.*

|  | **Total difficulties** | | | **Well-being** | | | **Self-efficacy** | | | **Self- Competence** | | | **Self-liking** | | | **Sleep problems** | | | **Help-seeking behavior** | | |
| --- | --- | --- | --- | --- | --- | --- | --- | --- | --- | --- | --- | --- | --- | --- | --- | --- | --- | --- | --- | --- | --- |
|  | F | *df* | *p* | F | *df* | *p* | F | *df* | *p* | F | *df* | *p* | F | *df* | *p* | F | *df* | *p* | F | *df* | *p* |
| Age | 2.95 | 1(788) | .086 | 0.48 | 1(787) | .489 | 1.27 | 1(769) | .261 | 1.30 | 1(786) | .255 | 0.08 | 1(786) | .779 | 0.56 | 1(787) | .454 | 3.30 | 1(784) | .070 |
| Sex | 17.20 | 1(788) | **<.001** | 39.58 | 1(787) | <.001 | 34.62 | 1(769) | **<.001** | 15.26 | 1(786) | **<.001** | 22.34 | 1(786) | **<.001** | 5.74 | 1(787) | .017 | 31.51 | 1(784) | <.001 |
| Time | 0.42 | 1(788) | .515 | 0.89 | 1(787) | .346 | 0.04 | 1(769) | .848 | 1.51 | 1(786) | .219 | 0.68 | 1(786) | .410 | 1.36 | 1(787) | .244 | 5.90 | 1(784) | .015 |
| Per protocol (pp) | 1.72 | 1(788) | .179 | 1.98 | 1(787) | .139 | 0.47 | 1(769) | .624 | 1.03 | 1(786) | .356 | 0.40 | 1(786) | .672 | 2.92 | 1(787) | .055 | 0.12 | 1(784) | .891 |
| Time*PP | 3.71 | 2(788) | **.025** | 0.31 | 2(787) | .733 | 0.22 | 2(769) | .800 | 1.87 | 2(786) | .155 | 2.23 | 2(786) | .108 | 0.79 | 2(787) | .457 | 0.20 | 2(784) | .818 |

*Note*. *P*-values that are <.05 are bolded.

**S2 Table**

*Results from Mixed Effect Models Predicting Mental Health, Well-being Self-Efficacy, Self-Competence, Self-liking, Sleep Problems and Help-seeking Behavior.*

|  | **Total difficulties** | | | **Well-being** | | | **Self-efficacy** | | | **Self- Competence** | | | **Self-liking** | | | **Sleep problems** | | | **Help-seeking behavior** | | |
| --- | --- | --- | --- | --- | --- | --- | --- | --- | --- | --- | --- | --- | --- | --- | --- | --- | --- | --- | --- | --- | --- |
|  | *B* | *S.E* | *p* | *B* | *S.E* | *p* | *B* | *S.E* | *p* | *B* | *S.E* | *p* | *B* | *S.E* | *p* | *B* | *S.E* | *p* | *B* | *S.E* | *p* |
| Age | -0.36 | 0.21 | .086 | 0.47 | 0.68 | .489 | 0.20 | 0.18 | .261 | 0.33 | 0.29 | .255 | 0.09 | 0.32 | .779 | -0.02 | 0.03 | .454 | 0.14 | 0.08 | .070 |
| Sex^a^ | -2.65 | 0.64 | **<.001** | 12.52 | 1.99 | **<.001** | 3.04 | 0.52 | **<.001** | 3.51 | 0.90 | **<.001** | 4.65 | 0.98 | **<.001** | -0.20 | 0.08 | **.017** | -1.33 | 0.24 | **<.001** |
| Time^b^ | 0.82 | 0.25 | **.001** | -0.22 | 1.23 | .861 | -0.21 | 0.34 | .536 | -0.85 | 0.42 | .044 | -0.34 | 0.40 | .395 | -0.04 | 0.05 | .409 | -0.44 | 0.18 | .**017** |
| Per protocol 1^c^ | -0.71 | 0.89 | .421 | 5.12 | 2.76 | .063 | 0.34 | 0.82 | .681 | 1.11 | 1.37 | .417 | 0.40 | 1.44 | .783 | 0.03 | 0.13 | .809 | 0.02 | 0.36 | .947 |
| Per protocol 2 ^c^ | -0.44 | 0.61 | .472 | 3.30 | 2.13 | .122 | 0.39 | 0.55 | .485 | 0.77 | 0.92 | .403 | 0.04 | 1.01 | .972 | -0.21 | 0.09 | **.019** | -0.11 | 0.25 | .660 |
| Time* per protocol 1 | -1.59 | 0.59 | **.007** | -1.99 | 2.64 | .450 | 0.34 | 0.52 | .508 | 1.37 | 0.74 | .066 | 1.63 | 0.79 | **.040** | -0.06 | 0.10 | .559 | 0.17 | 0.37 | .638 |
| Time* per protocol 2 | -0.43 | 0.36 | .235 | -0.08 | 1.69 | .964 | 0.18 | 0.43 | .673 | 0.09 | 0.65 | .888 | 0.13 | 0.60 | .824 | 0.06 | 0.07 | .422 | 0.15 | 0.26 | .574 |

*Note*. ^a^Males is the reference group, ^b^T1 is the reference group, ^c^per protocol 0 (control group) is the reference group. P-values that are <.05 are bolded.
